# A Correlation Study of Prognostic Risk Prediction for Colorectal Cancer Based on Autophagy Signature Genes

**DOI:** 10.3389/fonc.2021.595099

**Published:** 2021-05-25

**Authors:** Haibi Zhao, Chengzhi Huang, Yuwen Luo, Xiaoya Yao, Yong Hu, Muqing Wang, Xin Chen, Jun Zeng, Weixian Hu, Junjiang Wang, Rongjiang Li, Xueqing Yao

**Affiliations:** ^1^ School of Biology and Biological Engineering, South China University of Technology, Guangzhou, China; ^2^ Department of Gastrointestinal Surgery, Guangdong Provincial People’s Hospital, Guangdong Academy of Medical Sciences, Guangzhou, China; ^3^ Ganzhou Hospital (Ganzhou Municipal Hospital), Guangdong Provincial People's Hospital, Ganzhou, China; ^4^ School of Medicine, South China University of Technology, Guangzhou, China; ^5^ The Second School of Clinical Medicine, Southern Medical University, Guangzhou, China; ^6^ Medical College, Shantou University, Shantou, China; ^7^ Department of General Surgery, Baoan Central Hospital, The Fifth Affiliated Hospital of Shen Zhen University, Shen Zhen, China

**Keywords:** ARGs, prognostic indicator, CRC, nomogram, risk model

## Abstract

Autophagy plays a complex role in tumors, sometimes promoting cancer cell survival and sometimes inducing apoptosis, and its role in the colorectal tumor microenvironment is controversial. The purpose of this study was to investigate the prognostic value of autophagy-related genes (ARGs) in colorectal cancer. We identified 37 differentially expressed autophagy-related genes by collecting TCGA colorectal tumor transcriptome data. A single-factor COX regression equation was used to identify 11 key prognostic genes, and a prognostic risk prediction model was constructed based on multifactor COX analysis. We classified patients into high and low risk groups according to prognostic risk parameters (p <0.001) and determined the prognostic value they possessed by survival analysis and the receiver operating characteristic (ROC) curve in the training and test sets of internal tests. In a multifactorial independent prognostic analysis, this risk value could be used as an independent prognostic indicator (HR=1.167, 95% CI=1.078-1.264, P<0.001) and was a robust predictor without any staging interference. To make it more applicable to clinical procedures, we constructed nomogram based on risk parameters and parameters of key clinical characteristics. The area under ROC curve for 3-year and 5-year survival rates were 0.735 and 0.718, respectively. These will better enable us to monitor patient prognosis, thus improve patient outcomes.

## Introduction

Colorectal cancer (CRC) is one of the most common malignancies in the world. In 2018, there were nearly 881,000 deaths related to colorectal tumors (1). Current studies have shown that in addition to familial aggregation and hereditary CRC syndrome, colorectal cancer is associated with tissue inflammation, intestinal immune regulation, hormones, dietary habits, and intestinal flora composition (2).

Autophagy is an intracellular self-degradation process that can be stimulated under a variety of stressful conditions, such as organelle damage, protein abnormalities, and nutritional deficiencies. During autophagy, some cellular material is delivered to the lysosome for degradation in order to ensure the basic cellular functioning. In cancer, autophagy plays a dual role and its inhibition in advanced tumor stages may be an effective therapeutic approach, but targeting of autophagy still requires an understanding of its environmental and contextual dependence (3, 4). On the other hand, autophagy regulation is also important for the intestinal flora, and the interaction of this process with nuclear receptor signaling can modulate the inflammatory response (5). More importantly, autophagy also has a major impact on multidrug resistance after chemotherapy, and autophagy induced by anticancer drugs can activate apoptosis of drug-resistant cells, thereby reversing drug resistance (6).

Autophagy is known to be an important component of the integrative stress response, and Liu et al. (7) found that BRG1 affects colonic inflammation and tumors through autophagy-dependent oxidative stress isolation, suggesting that autophagy site could be a potential therapeutic target. In terms of drug therapy, Ping Jin et al. (8) found that autophagy inhibition enhanced the effect of ositinib-induced tumor cell apoptosis and growth inhibition. Thus, exploring molecular biomarkers of autophagy could help us understand more about the impact of autophagy in cancer, and could even be a way to discover new targets.

In our study, autophagy-related gene (ARG) expression profiles of colorectal cancer patients were obtained using The Cancer Genome Atlas (TCGA), prognostic impact genes were obtained by single-factor COX analysis, and a prognostic risk prediction model was constructed using multifactor COX minutes. The risk value is a characteristic parameter that allows us to robustly predict patient survival and to facilitate the clinical process, we have developed nomogram based on risk characteristic and clinical characteristics, which will help us to provide strong support for improving patient outcomes.

## Materials and Methods

### Data Sources

We downloaded FPKM data on gene expression of colorectal cancer transcripts from TCGA-GDC (https://portal.gdc.cancer.gov/) and obtained a total of 612 cases of colorectal cancer transcripts, including 44 normal samples and 568 tumor samples, as well as patient clinical data in XML format. HADb (http://www.autophagy.lu/clustering/) is a human autophagy public database that stores information on genes that have been reported to be associated with human autophagy. We obtained a total of 232 autophagy-related genes (ARGs) from HADb and extracted the expression of 232 ARGs from TCGA transcriptome data to obtain the autophagy-related gene expression matrix. The 363 samples from the GSE87211 dataset were used as the validation set. Since all data were collected from publicly available data in the HADb, GEO and TCGA databases, ethics committee approval was not required.

### Differential Expression ARGs Enrichment Analysis

We used R language for data analysis and extracted 222 autophagy-related genes expression profiles from the transcriptome data obtained by TCGA, and screened and evaluated whether they were differentially expressed in tumor and normal samples. SCREENING METHODS: Using the R language “Limma” package for data variance analysis. Wilcox test was used to identify differentially expressed ARGs, and 37 autophagy-differentiated genes were obtained by determining cut-off values based on FDR<0.05 and |log (FC)|>1 criterion. To obtain high-dimensional information, we used the enrichplot package of R and the ggplot2 package to visualize these different genes for GO analysis. The z-score method was used to obtain the cut-off values and the GOplot package was used to visualize the KEGG analysis to identify the main biological properties of these genes.

### Establishing a Risk Profile Associated With CRC Patient Survival

At the matching of TCGA transcriptomic tumor data with clinical data, by reducing some of the sample data with incomplete information, we obtained 540 cases and split them into a training set and a test set in a 7:3 ratios, with 378 case counts in the training set and 162 case counts in the test set. In the training set, we used single-factor Cox analysis to select ARGs that were significantly associated with the prognosis of CRC patients, multivariate Cox analysis to obtain the final prognostic ARGs, and established a prognostic model consisting of these genes. The prognostic model we constructed was based on a linear combination of relative expression levels of genes multiplied by regression coefficients, and the relative weights of the genes were represented in the multivariate Cox analysis, with the prognostic risk value as the final presented outcome. We used the median prognostic risk value as a risk cut-off value to classify CRC patients into high-risk and low-risk groups. To verify whether the prognostic risk value had a valid predictive efficiency, we combined the training and test sets and performed survival analysis and ROC curve analysis on the training set, test set and combined set, respectively. In addition, the GSE87211 dataset was downloaded from the GEO database as an external validation set, the risk score for each patient was calculated using the same formula as the training set, Kaplan-Meier curves were used to assess the predictive power of the model, and the expression levels of five key genes were examined in cancer and normal samples.

After determining that the risk parameter as an indicator already had predictive power, we further explored whether autophagy-related prognostic risk value could be used as an independent predictor of OS in the TCGA cohort of CRC patients.

We performed univariate Cox regression analysis and multivariate Cox regression analysis using the R language “ survival” package, and the characteristic of P<0.05 was considered significant for independent prognosis. In survival analysis, we used the “ survival” and “ survminer” software packages for survival analysis and picture plotting, and the Kaplan-Meier method was used to identify high and low risk groups by median, and the difference of P<0.05 was considered statistically significant. For the ROC curve analysis, we used the R language “survivalROC” package for the analysis and the Kaplan-Meier method for the 3-year ROC curve.

### GSEA Analysis and the Construction of Nomogram

We performed a GSEA enrichment analysis of the five key genes constituting the predicted risk values using GSEA 3.0 (http://www.broad.mit.edu/gsea/) and JAVA program (http://software.broadinstitute.org/gsea/downloads.jsp), after performing 1,000 permutations using the c2.cp.kegg.v7.4.symbols pathway gene set collection (containing 186 gene sets), and differences of P < 0.05 and FDR < 0.25 were considered statistically significant. To aid clinical procedures, we constructed nomogram combining risk profile parameters and clinic pathological risk factors as a quantitative predictive tool to assess clinical outcomes.

### Statistical Analysis

All statistical analyses, including single and multifactorial Cox regression analysis, survival analysis and ROC curve analysis, were performed using Rstudio (version 3.6.1). Quantitative data are shown as mean ± standard deviation, and statistical differences between the two groups were compared with Wilcox test. Heat maps, box line maps and forest maps were drawn using R. P < 0.05 was considered statistically significant.

## Results

### Differentially Expressed Autophagy-Related Genes

**Figure 1:**
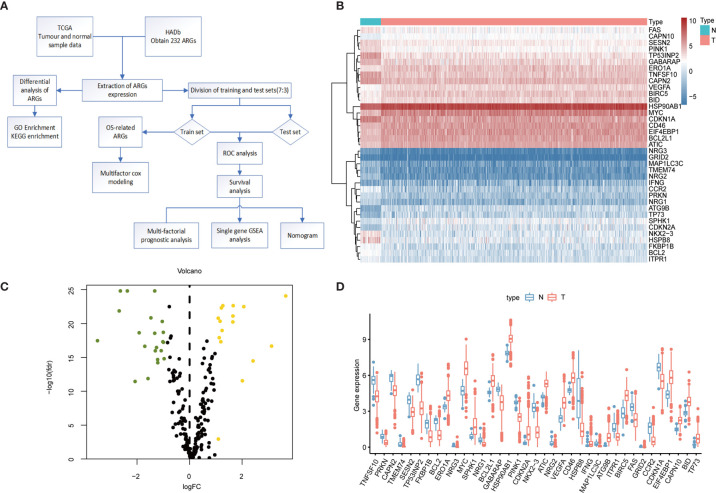
Differential expression of ARGs between colorectal cancer and normal tissues. **(A)** Research Flowchart. **(B)** Clustered heat map of expression levels of 37 autophagy differential genes. Green is normal tissue; Orange is tumor tissue. Red indicates high expression, blue indicates low expression. **(C)** Volcano diagram of ARGs expression, with green indicating low expression and yellow indicating high expression. **(D)** Expression of 37 ARGs in colorectal cancer tumor tissues and paired non-tumor samples. Red represents tumor samples and blue represents non-tumor samples. ARGs, Autophagy-Related Genes; FDR, false discovery rate; FC, Fold Change; TCGA, The Cancer Genome Atlas.

The flowchart of our designed study is shown in **Figure 1A**. We identified 37 differentially expressed genes from transcriptomic data of normal and tumor samples of the colorectal obtained from the TCGA database. The 21 genes that were significantly down-regulated in expression were HSPB8, NRG2, NKX2-3, TP53INP2, TMEM74, CCR2, NRG3, MAP1LC3C, BCL2, TNFSF10, PINK1, FKBP1B, PRKN, ITPR1, NRG1, FAS, GABARAP, GRID2. CAPN2, SESN2, and CDKN1A; the 16 genes whose expression was up-regulated were CAPN10, IFNG, BCL2L1, BID, ERO1A, ATIC, CD46, HSP90AB1, EIF4EBP1, BIRC5, VEGFA, SPHK1, MYC, TP73, CDKN2A, and ATG9B. In the heat map (**Figure 1B**) and box line plot (**Figure 1D**), we observed the expression of 37 genes in normal and tumor samples, while the volcano plots (**Figure 1C**) show the genetic screening.

### Functional Validation of Differential Autophagy-Related Genes

**Figure 2:**
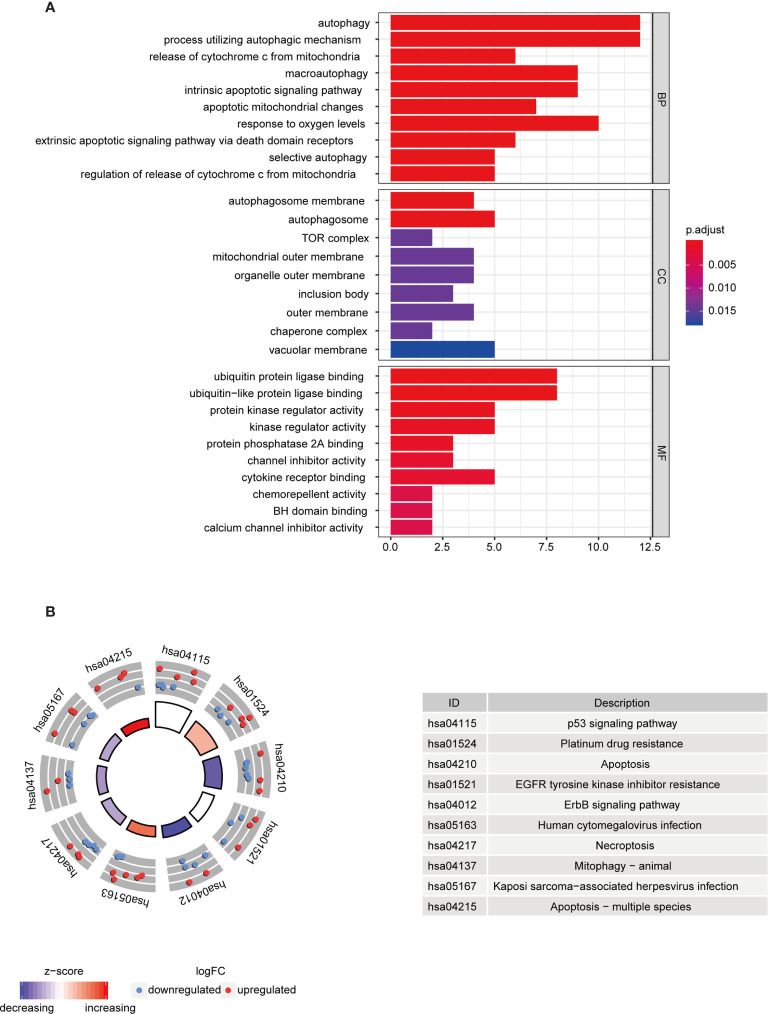
Functional analysis of differential autophagy genes. **(A)** GO analysis: top 10 gene functions for each category. Red end indicates high correlation; blue end indicates low correlation. BP, CC and MF, biological function, cellular component composition and molecular function. **(B)** KEGG analysis: top 10 gene enrichment pathways. Red circles indicate up-regulation and blue circles indicate downregulation. Different colors of the inner circles indicate the overall expression of genes clustered in this category. GO, Gene Ontology; KEGG, Kyoto Encyclopedia of Genes and Genomes.

To further understand the biological functions of differential autophagy genes, we performed GO and KEGG analyses on these genes. In the GO enrichment analysis, the biological functions of these 37 differential autophagy genes focused on the inherent regulation of apoptosis, oxygen content response, and muscle cartilage changes, in addition to the regulation of cellular autophagy. They mainly play a role in the composition of cellular components such as autophagosome membranes, autophagosomes, and complex TOR functions. In molecular functions they mainly play the role of ubiquitin protein ligase binding, ubiquitin-like protein ligase binding, protein kinase regulator activity, etc. (**Figure 2A**). We learned from the KEGG analysis that these genes are mainly involved in the regulation of p53 signaling pathway, albumin resistance, apoptosis, EGFR tyrosine kinase inhibitor resistance, ErbB signaling pathway, and other signaling pathways (**Figure 2B**).

### Autophagy Gene Prognosis Analysis and Risk Model Construction

**Figure 3:**
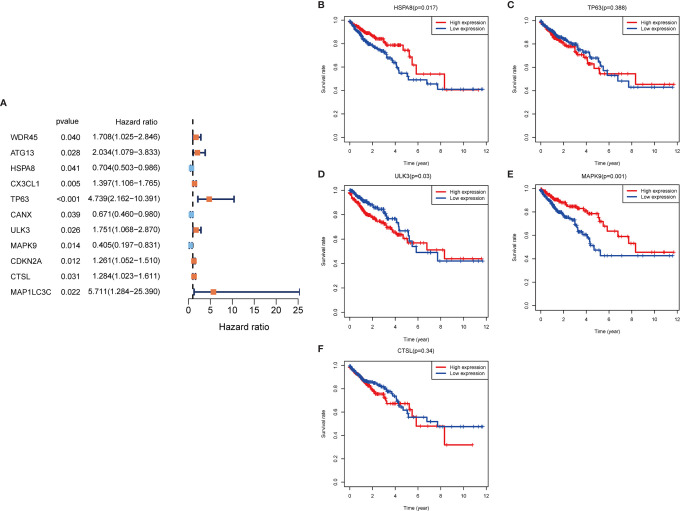
Autophagy gene prognosis analysis. **(A)** Forest plot: Univariate COX regression analysis yielded prognosis-related genes, high risk genes: HR>1, low risk genes: HR<1. **(B–F)** Survival analysis of HSPA8, TP63, ULK3, MAPK9 and CTSL genes. The HSPA8 and MAPK9 high expression groups had better survival rates, while the other genes were not significantly different. HR, Hazard Ratio.

In the training set sample, we assessed the relationship between 232 autophagy-related genes and overall survival (OS) by univariate Cox regression analysis (**Figure 3A**), yielding 11 prognosis-related genes, including three low-risk genes: HSPA8, CANX, and MAPK9; and eight high-risk genes: WDR45, ATG13, CX3CL1, TP63, ULK3, CDKN2A, CTSL, and MAP1LC3C.

To determine whether these OS-related genes act non-independently, we performed a multifactorial COX analysis of these 11 genes to identify the characteristic parameters that could truly influence OS. In the multifactorial COX analysis, we identified five genes that were used to construct the risk model: HSPA8, TP63, ULK3, MAPK9, and CTSL. We used these five genes to construct the prognostic prediction model: (-0.5319×HSPA8 expression value) + (1.4333×TP63 expression value) + (0.5014×ULK3 expression value) + (-0.7018×MAPK9 expression value) + (0.3298×CTSL expression value) = patient risk value. HSPA8 and MAPK9 were low-risk genes, and TP63, ULK3, and CTSL were high-risk genes (**Table 1**). To gain a better understanding of these five genes, we performed survival analysis on them (**Figures 3B–F**).

**Table 1:** Risk prognosis model table. HSPA8 and MAPK9 are low-risk genes and TP63, ULK3, and CTSL are high-risk genes.

ID	Coef	HR	HR.95L	HR.95H	P value
HSPA8	-0.53187368	0.587503146	0.408231369	0.845500796	0.004189771
TP63	1.433268924	4.192381391	1.804266321	9.741389908	0.000862622
ULK3	0.50141811	1.651060998	0.99830923	2.730619268	0.050775569
MAPK9	-0.70179156	0.495696439	0.234641897	1.047191327	0.065897061
CTSL	0.329839978	1.390745561	1.076044679	1.797484114	0.011738924

### Validation of Risk Parameters

**Figure 4:**
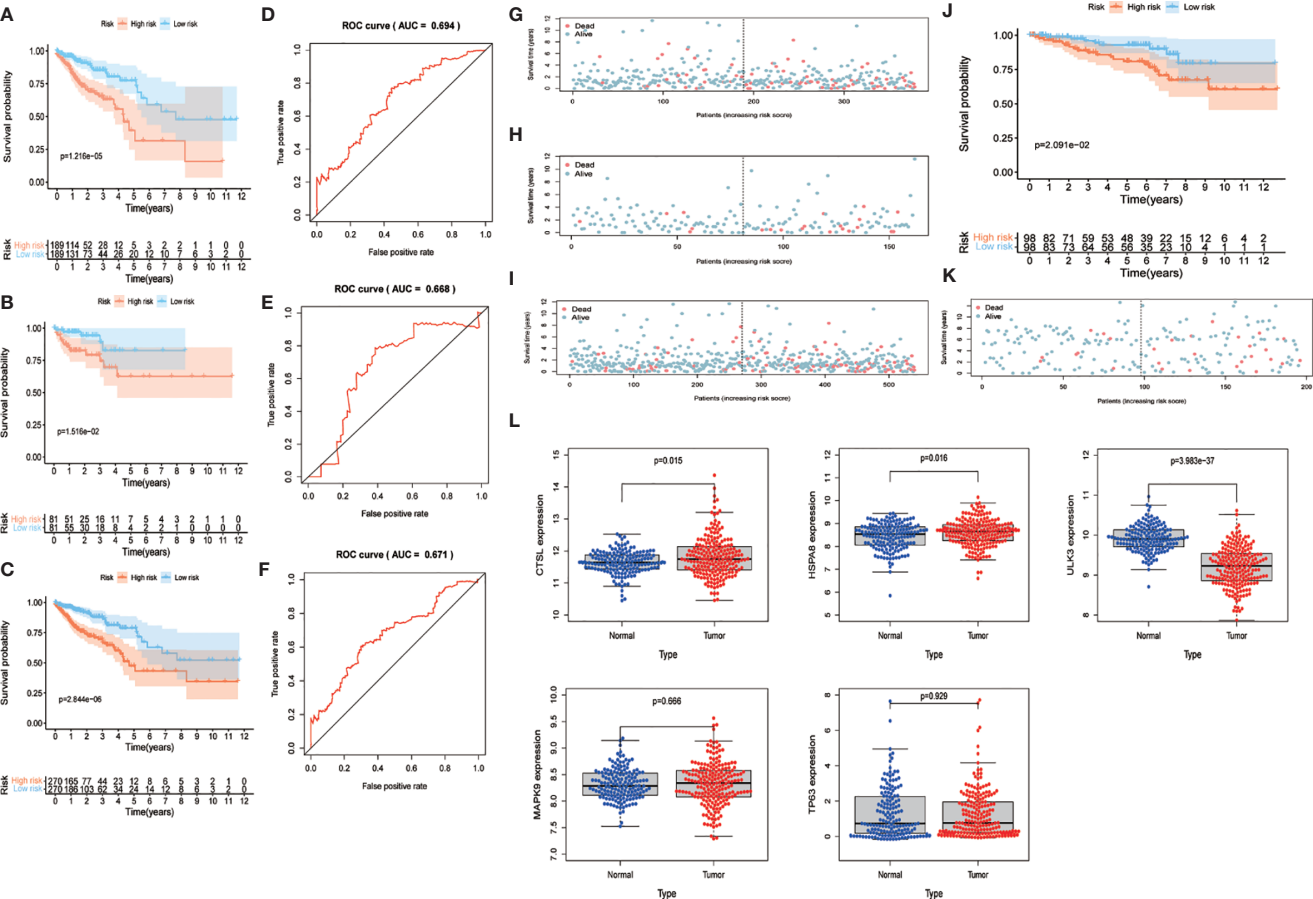
Validation of risk parameters. **(A–C)** Training set, test set and combined set survival curves. Kaplan-Meier plots indicate the survival status of patients in the high and low risk groups, with shorter overall survival time in the high risk group compared to the low risk group. **(D–E)** Training set, test set and combined set ROC curves with AUC=0.694, AUC=0.668 and AUC=0.671. **(G–I)** Survival status of training set, test set and combined set. Death cases in high and low risk groups are shown in blue for survival and red for death. **(J)** GSE87211 survival curves. The lowrisk group has better OS performance. **(K)** GSE87211 survival status. **(L)** Expression of 5 genes in normal and tumor samples. OS, overall survival; ROC, receiver operating characteristic; AUC, The area under the curve.

We combined the training and test sets and calculated the risk values for each patient in the 3 sets, categorized patients into high- and low-risk groups according to the median, and analyzed them for OS to see if the predicted risk values were significant. The results showed that both in the training set (**Figure 4A**), the test set (**Figure 4B**) and merged set (**Figure 4C**), the low-risk patients had better OS. Similarly, in the observation of patient survival status, the number of deaths increased as the patient’s risk value increased, and both the training set (**Figure 4G**), the test set (**Figure 4H**) and merged set (**Figure 4I**) were significant for the number of deaths on the side with the highest risk value. In addition, to test the stability predictive ability of the risk parameter, we performed ROC analysis in the training set (**Figure 4D**) , the test set (**Figure 4E**) and merged set **Figure 4F**) with the area under the curve of AUC=0.694, AUC=0.668 and AUC=0.671, respectively.

To further validate the predictive power of the risk parameters, the risk scores of patients were calculated in GSE87211 using the same formula, and patients were divided into high-risk and low-risk groups according to the median risk score. In the validation set, the trends of the distribution of survival curves (**Figure 4J**) and survival status (**Figure 4K**) of patients were similar to the trends in the training set, and OS was significantly lower in the high-risk group (P <.001) (**Figure 4J**). Meanwhile, we extracted five key gene expressions in this set to detect the expression trends of these genes in normal and tumor samples (**Figure 4L**), and the differences were considered statistically significant in CTSL (P = 0.015), HAPA8 (P = 0.016), and ULK3 (P < 0.01), while MAPK9 (P = 0.666) and TP63 (P = 0.929) were not considered significant. Also, inconsistent with the previous results, we previously found TP63, ULK3 and CTSL to be high-risk genes, whereas in this pooled result, TP63 appears to be a low-risk gene and ULK3 and CTSL continue to be high-risk genes.

### Prognostic Value of Risk Parameters

**Figure 5:**
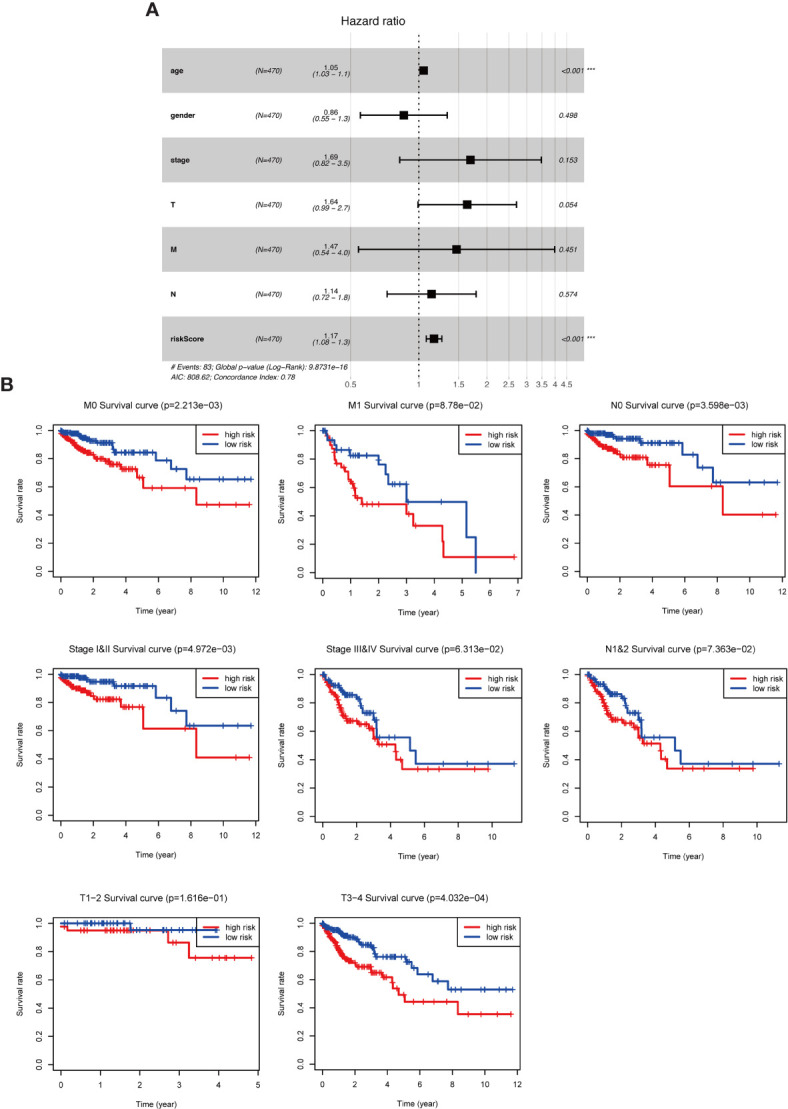
The role of risk parameters in predicting the prognosis of patients with colorectal cancer. **(A)** Multifactor independent prognostic analysis. Age and risk value had a statistically significant effect on prognosis, p<0.001. **(B)** Survival analysis of pathological parameters based on risk values. In the survival analysis of patients at M0, M1, N0, N1&2, Stage I&II, Stage III&IV, T1-2, T3-4, the low-risk group had a more significant survival rate.

To further assess the role of the risk parameter in predicting the prognosis of patients with colorectal cancer, we extracted the clinic pathological characteristics of patients’ age, sex, Stage and TNM stage and performed a multifactorial independent prognostic analysis with them and risk parameter. We found that risk parameter (HR = 1.167, 95% CI = 1.078-1.264, P < 0.001) and age (HR = 1.051, 95% CI = 1.028-1.073, P < 0.001) could be used as prognostic parameter in the multifactorial analysis of colorectal cancer patients. Independent prognostic indicator for patients with colorectal cancer (**Figure 5A**). This result confirms that the risk parameter as an indicator will be independent of other clinic pathological characteristics and that stable predictions can be obtained. Next, we stratified patients according to Stage, T stage, N stage and M stage to examine the prognostic value of risk parameter for different grades. The ability to predict survival in a high- and low-risk group of patients based on the risk parameter of the autophagy signature genes was not affected by any staging (**Figure 5B**).

### GSEA Analysis of 5 Genes

**Figure 6:**
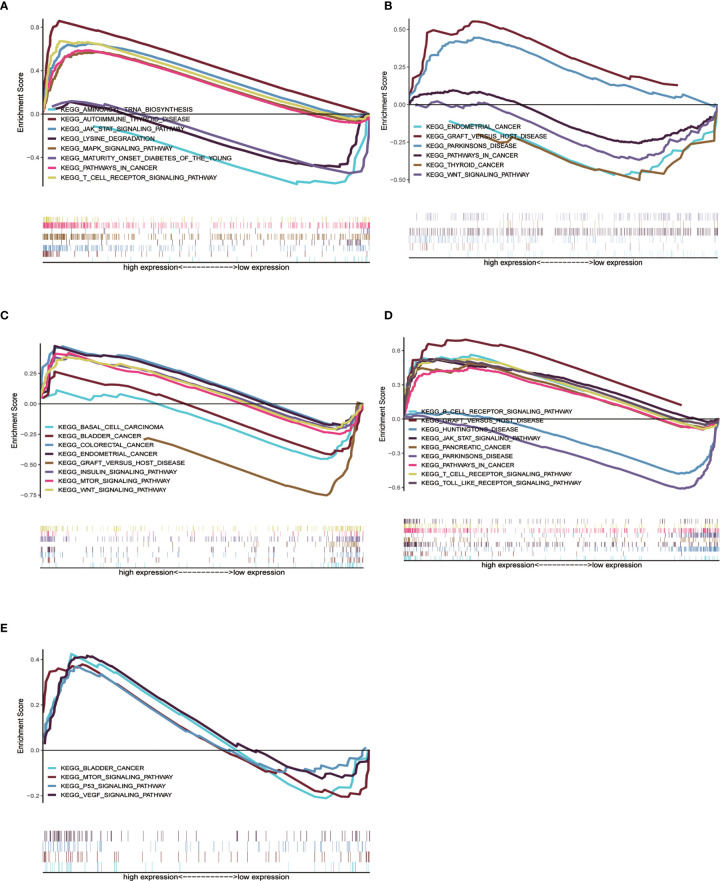
Single gene GSEA analysis of 5 genes. **(A–E)** High and low expression groups of CTSL, HSPA8, MAPK9, TP63 and ULK3 genes, respectively, showed enrichment in the KEGG pathway associated with cancer.

We already understand the significance of the risk parameter for prognosis, but are there certain pathways in which the key genes that make up this parameter also influence tumor development? To answer this query, we performed GSEA analysis on each of these five key genes to observe their high and low expression groups on the KEGG pathway, and we focused on observing the relationship between these genes and the cancer pathway. In CTSL, its high expression was involved in several cancer pathways (**Figure 6A**), such as JAK signaling pathway and cancer signaling pathway (**Supplementary Table 1**). High and low expression of HSPA8 was closely associated with the development of multiple cancer pathways (**Figure 6B**), such as the WNT signaling pathway, thyroid cancer and Parkinson’s disease (**Supplementary Table 2**). High and low MAPK9 expression is also involved in multiple cancer pathways (**Figure 6C**), such as colorectal and bladder cancers (**Supplementary Table 3**) .High and low TP63 expression is involved in various diseases such as Parkinson’s disease and pancreatic cancer (**Figure 6D** and **Supplementary Table 4**). High expression of ULK3 is involved in bladder cancer and MTOR signaling pathway, among others (**Figure 6E** and **Supplementary Table 5**). In short, the results of GSEA analysis imply that these genes are associated with the development and progression of tumors.

### Create and Validate Nomogram

**Figure 7:**
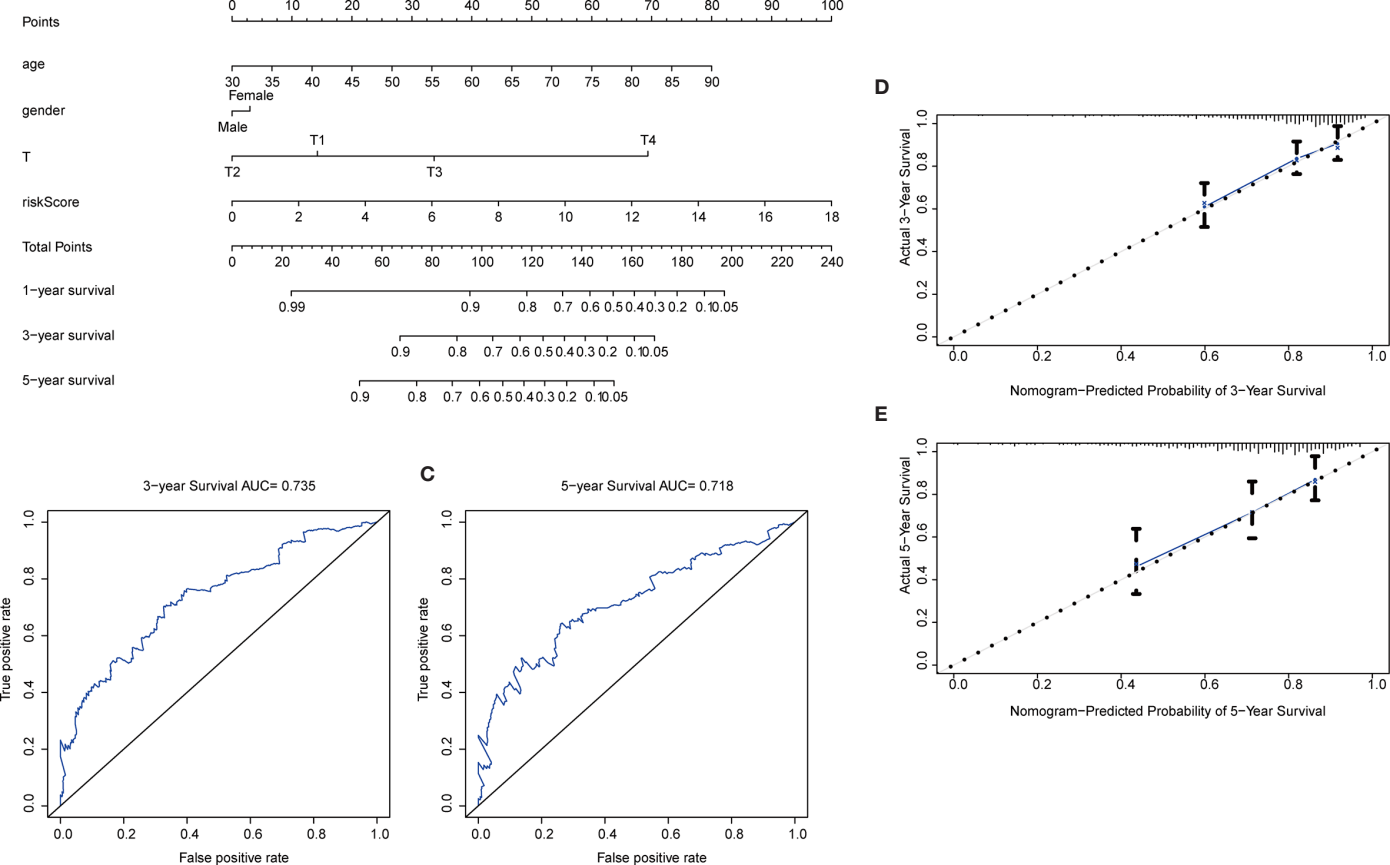
Nomogram construction. **(A)** Nomogram. Predictive characteristic factors consisted of sex, age, T-stage and risk parameters. **(B–C)** Time-dependent ROC curves. Assessment of model accuracy, 3-year AUC = 0.735 and 5-year AUC = 0.718. **(D–E)** Nomogram calibration curves. 3-year and 5-year calibration curves are close to the standard curve.

To further extend the applicability of the risk parameter, we combined the risk parameter with three clinical characteristics: sex, age and T stage to construct nomogram, which can directly predict the survival status of CRC patients. Survival of CRC patients at 1, 3, and 5 years was predicted by calculating the total nomogram score (**Figure 7A**). We applied ROC curves to assess the accuracy of this scoring system, with a 3-year predicted AUC of 0.735 (**Figure 7B**) and a 5-year predicted AUC of 0.718 (**Figure 7C**). This suggests that the Nomogram prediction model we developed is of high value for the postoperative prognosis of CRC patients. In addition, in the Nomogram calibration curves, both the 3-year (**Figure 7D**) and 5-year (**Figure 7E**) calibration curves are close to the reference line.

### Prognostic Model for All Genes

**Figure 8:**
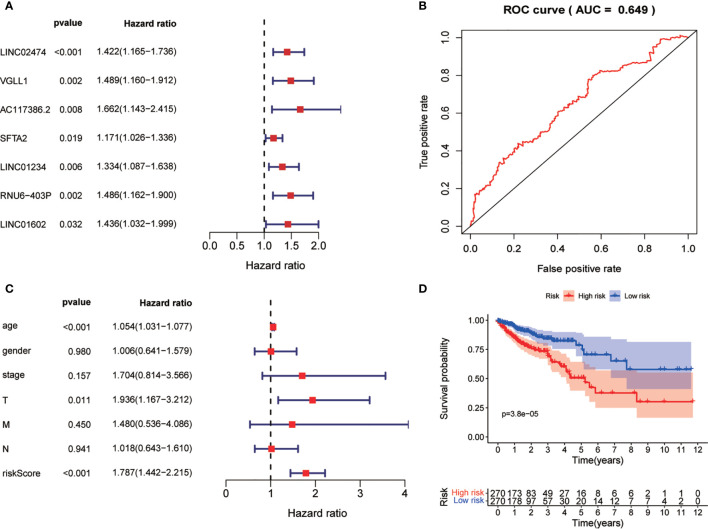
Non-ARG gene prediction model. **(A)** Forest plot of Univariate COX analysis. **(B)** Forest plot of multi-factor independent prognostic analysis. **(C)** Non-ARG gene prediction model ROC validation curve. **(D)** Survival curves.

After obtaining the autophagy-related gene prognostic model, we screened all genes with the aim of constructing a non-ARG prognostic risk model for comparison with existing models. We used the method described previously for constructing the autophagy-related gene prognostic model to construct a new model in which differential gene screening we filtered according to |log FC|>7, FDR<0.05 and obtained 43 differential genes. After performing univariate COX regression analysis on these differential genes, we obtained 7 prognosis-related genes: LINC02474, VGLL1, AC117386.2, SFTA2, LINC01234, RNU6-403P, LINC01602 (**Figures 8A, B**). Finally, this model was validated by survival analysis and ROC curves (**Figures 8C, D**). Of interest to us, the ROC curve of this all differential gene prognostic model with AUC=0.649 was not better than the model we constructed with autophagy-related genes (AUC=0.694).

## Discussion

Colorectal cancer is one of the world’s deadliest cancers, and although new treatments have been developed to increase the overall survival of advanced patients, improving early detection can better reduce the incidence and mortality of colorectal cancer because it only causes symptoms in the middle and late stages. Currently, targeted therapy is a new approach in the treatment of colorectal cancer and has been successful in prolonging the overall survival of CRC patients (1). And in the direction of molecular targeting, the development of potential biomarkers not only improves the early detection rate of CRC, but is also necessary for the development of drugs that can improve patient survival (9, 10). Autophagy has been found to play an important role in cancer development and has been explored as a potential therapeutic target in a variety of malignancies (11). And because of the complex role that autophagy has in cancer, it makes deciphering autophagy crucial (12). In colorectal cancer, inhibition of autophagy has been found to be a promising therapeutic strategy to increase the cytotoxicity of chemotherapeutic agents, and inhibition of autophagy through the use of digitizing can sensitize CRC cells to 5-fluorouracil, significantly reducing the viability of cancer cells (13). In most of the previous studies, autophagy was mainly explored with autophagy signaling pathways or signaling genes, and autophagy genes themselves were less studied, so we wanted to link autophagy-related genes to colorectal cancer and seek the impact of autophagy-related genes on the prognosis of colorectal cancer patients, and these genes will provide new possibilities to improve the treatment and prognosis of colorectal cancer. We screened and identified key prognostic ARGs from autophagy-related genes and developed a risk prediction model based on these genes, and patients in the high-risk group were strongly associated with poor prognosis.

In this study, we dug deeper into the TCGA database to analyze the expression profile of ARGs using its transcriptomic data, aiming to find suitable molecular markers for predicting the prognosis of colorectal cancer patients. First, we screened for 37 differentially expressed ARGs between colorectal tumors and non-tumor tissues. Second, to better understand the function of these genes in CRC, we performed GO and KEGG analyses on them. Notably, in the KEGG analysis, these genes were mainly enriched in the p53 signaling pathway, platinum resistance and apoptosis pathway. In a previous report, the initiation of autophagy in sorafenib-resistant hepatocellular carcinoma cells enhanced the resistance of cancer cells to sorafenib (14). In addition, it has also been found that when autophagy dies, it reduces the proliferation and migration of lung adenocarcinoma cells to the extent that reducing increased tumor autophagy may be an effective therapeutic strategy (15). Based on these findings, we speculate that ARGs play a multifaceted effect in cancer. To further understand the role these genes play in colorectal cancer, we divided the TCGA data into a training set and a test set. In the training set we performed a single factor COX regression analysis and obtained 11 autophagy-related genes that were associated with prognosis. In the multivariate COX analysis, we obtained five key genes that had independent effects on patient prognosis without interference from other factors, namely HSPA8, TP63, ULK3, MAPK9, and CTSL. Using these prognostic genes we developed a prognostic risk model, and the risk parameter obtained may be used as an independent prognostic indicator for CRC patients. Subsequently, we identified a significant correlation between this risk value and prognosis through a multifactorial independent prognostic analysis. To test the reliability of this risk model, we further clarified the usability of this model by performing survival and ROC analyses on them in the training and test sets. The risk value is a stable predictor regardless of the clinical stage. Finally, we have extended this model by developing nomogram so that it can be more clinically applicable. The good level of prediction in the time-dependent ROC curves and calibration curves was demonstrated in our developed nomogram with sex, age, T-stage and risk parameter as test parameters, proving that this nomogram can effectively assess patient prognosis.

The five key genes obtained in our study, HSPA8 and MAPK9 were low-risk genes and TP63, ULK3, and CTSL were high-risk genes. In a previous study, HSPA8 was found to be important for glioblastoma, and knockdown of HSPA8 interferes with the tumorigenic properties of glioblastoma cells ectopically overexpressing nesting proteins (16). In gastric cancer, HSPA8 interacts with GKN2 to promote oxidative stress-induced apoptosis, inhibit the NF-κB signaling pathway, and activate the JNK signaling pathway (17). MAPK9 is a member of the MAP kinase family and acts as an integration point for a variety of biochemical signals involved in various cellular processes such as proliferation, differentiation, transcriptional regulation and development. It has been found that MAPK8/9 has a non-essential role in starvation-induced autophagy and that its regulated gene expression may lead to an increase in autophagy, but may lead to a decrease in autophagy under different circumstances (18). MAPK8 also known as c-Jun N-terminal kinase, is a key factor in JNK activation, which generates anti-apoptotic signals during the initial phase of JNK activation in the early stages of the endoplasmic reticulum stress response (19). The transcription factor TP63 is a member of the p53 family and plays a key role in epidermal development. In the development of squamous cell carcinoma, TP63 plays an important role in chromatin remodeling and enhancer reprogramming and epidermal differentiation (20). In esophageal squamous cell carcinoma, Jiang et al. (21) found that TP63, SOX2 and KLF5 are part of a core regulatory network that determines cellular chromatin accessibility, epigenetic modifications and gene expression patterns. ADUK, the orthologue of Drosophila Ulk3, is an autophagy-induced Atg1 independent pathway. Loss of ADUK attenuates the autophagy response to complex stressors, whereas it has no effect on the induction of autophagy in response to known Atg1-dependent stimuli (22). In squamous cell carcinoma, inhibition of the ULK3 gene inhibits fibroblast effector gene expression as well as GLI2 activation, while inhibiting the growth-enhancing and oncogenic properties of these cells of neighboring cancer cells (23). CTSL is a lysosomal cysteine protease that plays a major role in the metabolism of intracellular proteolysis. CTSL can contribute to ionizing radiation-induced EMT in lung cancer through the mut-p53/Egr-1 signaling pathway, and the expression level of CTSL is significantly higher in tumor tissues than in adjacent tissues, positively correlating with the grade of the tumor (24). In the study by Mao et al. (25) CTSL was significantly associated with autophagy and played a key role in degrading the extracellular matrix to promote metastasis.

Currently, there have been significant advances in the development of public databases, and an increasing number of expression profiling-based studies have been generated with the support of public databases, such as Qiu et al. (26) using the TCGA and GEO public databases to obtain seven immune-related genes that could help provide potential therapeutic targets for bladder cancer. Wang et al. (27) established an autophagy-associated multi-gene expression signature network, which provides direction for the individualized prognosis of glioblastoma patients. Our research focuses on the link between molecular biomarkers and clinical signature parameters so that these prognostic parameters can be translated into the clinic. However, our study also has some limitations, being a retrospective study based on TCGA data with a limited number of cases and clinical characteristic parameters available, so more prognostic variables are not yet found in relation to risk indices, and these will need to be determined by further studies.

## Conclusion

In conclusion, by mining the TCGA database ARGs expression profile, we constructed a risk scoring model and identified risk parameter value with independent prognostic value, and this risk value can help us effectively predict the survival status of colorectal cancer patients. We have also developed a nomogram for predicting patient survival index, which will provide strong support for assessing patient prognosis.

## Data Availability Statement

Publicly available datasets were analyzed in this study. This data can be found here: TCGA-GDC (https://portal.gdc.cancer.gov/) https://www.ncbi.nlm.nih.gov/geo/query/acc.cgi?acc=GSE87211.

## Author Contributions

HZ, CH, YL, and XueY presented the concept of this study. HZ, CH, YL, and XiaoY obtained the data and performed data collation and analysis. HZ, YH, and MW performed the statistical analysis. XC, JZ, WH, and JW provided suggestions for statistical methods. YW, XiaoY, and RL provided suggestions for graphical treatment. HZ wrote the first draft of the manuscript. CH, YL, XueY, and XiaoY revised the manuscript. All authors contributed to the article and approved the submitted version.

## Funding

This work was supported by grants from the Science and Technology Planning Project of Guangdong Province, China (No. 2017A030223006, 2016A020215128), the Science and Technology Planning Project of Guangzhou, China (No. 201704020077), the Second Batch of Scientific Research Projects of Dengfeng Plan (NO. DFJH201913), the Research Fund of CSCO-Roche Oncology (NO. Y-2019Roche-190) and the Research Fund of CSCO-Hansoh Oncology (NO. Y-HS2019/2-050), the Research Fund of Guangdong General Hospital (No. y012015338), the Yuexiu Science and Information Center of Guangzhou Scientific Foundation (No. 2012-GX-046).

## Conflict of Interest

The authors declare that the research was conducted in the absence of any commercial or financial relationships that could be construed as a potential conflict of interest.

## Supplementary Material

The SupplementaryMaterial for this article can be found online at:https://www.frontiersin.org/articles/10.3389/fonc.2021.595099/full#supplementary-material
